# Pelvic packing – status 2024

**DOI:** 10.1007/s00402-024-05699-3

**Published:** 2025-01-11

**Authors:** Axel Gänsslen, Tim Pohlemann, Jan Lindahl, Jan Erik Madsen

**Affiliations:** 1 Department of Trauma Surgery, Hannover Medical School, Carl-Neuberg-Str. 1, 30625 Hannover, Germany; 2 University Hospital, Johannes Wesling Hospital, Hans-Nolte-Straße 1, 32429 Minden, Germany; 3 Department of Trauma, Hand and Reconstructive Surgery, University of Homburg, Homburg, Saar Germany; 4 Department of Orthopaedics and Traumatology, Pelvis and Lower Extremity, Orthopaedic and Trauma Surgery Unit, Helsinki University Hospital and University of Helsinki, Helsinki, Finland; 5 Division of Orthopaedic Surgery, Oslo University Hospital, Oslo, Norway; 6 Institute of Clinical Medicine, Faculty of Medicine, University of Oslo, Oslo, Norway

**Keywords:** Historical development, Pelvic packing, State of the art, Results, Complications, Controversies

## Abstract

Patients with unstable hemodynamics and unstable pelvic ring injuries are still demanding patients regarding initial treatment and survival. Several concepts were reported during the last 30 years. Mechanical stabilization of the pelvis together with hemorrhage control offer the best treatment option in these patients. While pelvic ring stabilization using pelvic binders, external fixators and the pelvic C-clamp are the basis for mechanical stability of the pelvic ring, the optimal modality for pelvic bleeding control is still under discussion. Beside angioembolization (AE) and Resuscitative Endovascular Balloon Occlusion of the Aorta (REBOA), pelvic packing PP (predominantly extraperitoneal) with direct access to the pelvic bleeding sources, are potential options. The present overview represents the present status, results and the value of pelvic packing in treating these patients. Interpretation of these results must consider the difference between the initial European concept of pelvic ring stabilization followed by PP in contrast to the North American concept with a reduced rate of pelvic ring stabilizations.

## Introduction

Historically, tamponading of bleeding wounds became one of the standard procedures for hemorrhage control.

„Die Anfänge der Chirurgie sind gleich denen der Heilkunde überhaupt in sagenhaftes Dunkel gehüllt. Nächst rohen Hülfsleistungen bei der Entbindung entwickeln sich von allen Theilen der ärztlichen Thätigkeit unzweifelhaft am frühesten die einfachen chirurgischen Manipulationen: Entfernung eingedrungener Geschosse, Stillung von Blutungen durch mechanische Mittel, kaltes Wasser u.s.w., Zurückführung verrenkter und gebrochener Glieder in ihre normale Lage und Befestigung in derselben durch einfache Verbände“ [32].

“The begin of surgery, like those of medicine in general, are wrapped in legendary darkness. Of all the parts of medical activity, simple surgical manipulations are undoubtedly the earliest to develop next to aids during childbirth: removal of penetrated bullets, stopping bleeding by mechanical means, cold water, etc., reduction of dislocated and broken limbs to their normal position and fixing them by simple bandages” [1].

Already in the 10th century, the Arabian surgeon Abu al-Qasim Khalaf ibn al-Abbas al-Zahrawi (Latin Abulcasis) (936–1013), known as al-Zahrawi developed special medical instruments which were used for cauterizing of arteries and he also introduced the use of a ligature to control bleeding from arteries [55].

In the 14th century, Guy de Chauliac in France described five treatment modalities for hemorrhage control [64]:

- Suture of wounds.
- Tamponade.
- Vein compression.
- Arterial ligation.
- Cauterization.

Jean-Louis Petit introduced the concept of the hemostatic tourniquet in the early 18th century for bleeding control in extremity amputation [35, 54].

In both millenniums AC, tamponading bleeding wounds was considered an option for hemorrhage control.

In the following, the historical development of truncal hemostasis and hemostasis after pelvic trauma including early ideas of surgical intrapelvic hemorrhage control are presented followed by the current results after pelvic tamponade.

## Lessons learned in trunk hemorrhage control in the 20th century

Packing of bleeding wounds was still a well-known treatment modality [46].

Abdominal packing was introduced especially for severe intraabdominal bleeding, e.g. liver injury as a temporary treatment option in patients in extremis. This method was accepted for patients in a pathophysiological state, which does not allow a complete definitive treatment, most often when acidosis, coagulopathy and hypothermia (lethal triad of death) were present [48].

Hepatic packing, as a prototype injury, to control hemorrhage was already established in the 1890s and early 1900s, while perihepatic packing was introduced in the late 1920s [22]. Due to the risk of re-bleeding and infection, this concept was critically discussed for decades, until in the 1980ies patients with major hepatic trauma and accompanying parameters of the lethal triad of death were identified to benefit from this approach, with increasing survival rates [10, 13, 23, 40, 79].

In 1983 trauma laparotomy was indicated in trauma patients with relevant coagulopathy and intraabdominal injuries and included temporary intraabdominal packing, selected blood-vessel ligation or repair, quick bowel resection without anastomosis, and selected biliopancreatic drainage, while definitive treatment was performed when normal coagulation had recovered [78].

Scalea definded the principles of damage control treatment in these few patients [75]:

- only blood loss kills early.
- GI injuries cause problems much later.
- everything takes longer than you think.
- it’s easy to miss an injury if you rush.
- hypothermia, acidosis, and coagulopathy only lead to more of the same.
- the best place for a sick person is in the ICU.

This prioritized damage control concept for abdominal injuries lead the sickest patient paradoxically to have the least number of definitive procedures to break the lethal triad of death cycle [48].

Routine four quadrant packing became the standard of care in severely injured patients with intraabdominal bleeding [1, 52].

Rotondo et al. divided the damage control laparotomy into three phases [73]:

- abbreviated index operation focused on rapid surgical hemorrhage control and control of contamination followed by intra-abdominal packing and temporary closure.
- ICU resuscitation to correct hypothermia, coagulopathy, and acidosis.
- re-exploration for definitive surgical repair.

Lesson learned from general surgery was an abbreviated approach to the bleeding abdomen with packing of hemorrhage and contamination control.

## Focussing on the pelvis–early ideas

Peltier stated from his experience, that “retroperitoneal, intrapelvic hemorrhage” should be treated “by rapid and continuing blood replacement without opening the peritoneum or draining the hematoma” (Peltier 1965), while Fleming et al. discussed retroperitoneal opening in pelvic bleeding. The literature agreed in opening to control expanding arterial hematomas, in venous hemorrhage opening of the peritoneum may prevent a potential tamponade effect [3, 24, 86].

It was already mentioned that bleeding predominantly occurs from the fracture zone [24]. Bleeding from fractures sites, muscles and vessels may lead to relevant retroperitoneal hematoma [24, 33]. Fleming reported on isolated transfusion to patients with pelvic bleeding but presented a mortality rate of 75% [24]. For a long period of time isolated transfusion treatment was performed with the idea to generate a tamponade effect [25, 56].

Opening of the retroperitoneum to control pelvic bleeding was thought to be associated with potential torrential bleeding ( [6, 67].

During the 70ies and 80ies blood replacement was the primary option in treating pelvic retroperitoneal bleeding, due to fear of preventing a tamponade effect with opening of the peritoneum.

In most patients with stable pelvic ring injuries and stable vital signs, bleeding into the closed pelvic space will be self-limiting [18]. Due to the so-called “chimney effect”, a self-tamponade is prevented in highly unstable pelvic ring disruptions [85] as traumatic destruction of fascia, septa, muscle compartments and bony or ligamentous abutment avoids adequate compression [85]. This retroperitoneal pelvic bleeding was already described by Hubbard et al. for type C injuries of the pelvis [38] .


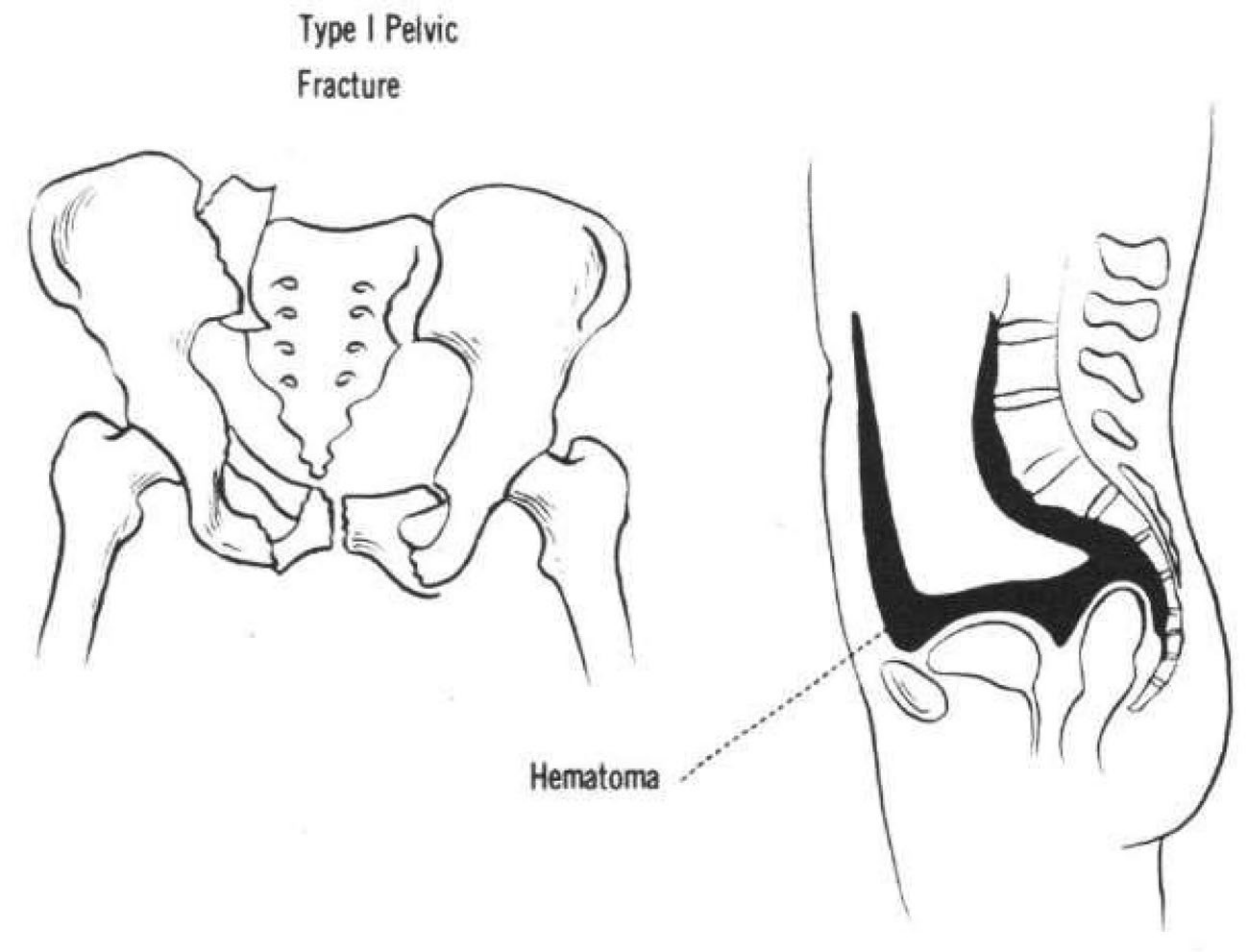


In unstable pelvic ring injuries, the musculo-fascial corridors are often completely disrupted, leading to unrestricted bleeding within the retroperitoneal space (“chimney effect”).

Grimm et al. found in an experimental study, that even after infusion of 20 L into the retroperitoneal space external fixation led to increased pressures of 9mmHg [31].

This can lead to uncontrolled hemorrhage creeping cranially along the psoas muscle or along the gluteal muscles, with the risk of exsanguination or pelvic and abdominal compartment syndromes [27].

Because the retroperitoneum is not a closed space, hemorrhagic pressure-induced tamponade cannot be expected [31, 85].

Consequently, in the early and mid 70ies the problem of venous pelvic hemorrhage was discussed, and tamponade treatment was recommended by experts without data on patients.

Reynolds and Balsano noted that the veins and venous plexus are closer to the pelvic bone, with a very thin wall compared to arteries making them at risk for injury. Tamponading these bleedings may be advantageous [69]. In 1976 Kimball Maull et al. proposed angiography to control arterial bleeding and recommended tamponade for venous bleeding [56]. Flint et al. reported on 10% retroperitoneal packing for pelvic bleeding control in a group of hemodynamically unstable patients with pelvic (crush) fractures, without describing their technique [25].

In the 70ies pelvic packing was introduced as an option in fracture related pelvic bleeding.

### Stabilization of the pelvis–early ideas

Peltier reported in 1965 that trauma laparotomy was often performed in patients with pelvic fractures/injuries without detecting intraabdominal organ injuries or bleeding sources (“Blood may be found in the abdominal cavity without aim intra-abdomiminal bleeding point.”) [63]. From the laparotomy approach, retroperitoneal bleeding often could not be controlled. Another problem arose from the orthopaedic surgeon, who neglected stabilization of the pelvis with resulting on-going bleeding [63]. Peltier clearly recommended, that “a higher priority to the stabilization of pelvic fractures” is necessary.

Immobilization of pelvic fractures was thought to be of relevance by bed rest, pelvic slings with or without additional skeletal traction, and bilateral limb turnbuckle casts [6]. In 1981, Brotman stated that “Theoretically, reduction and early stabilization are beneficial in terms of decreasing blood loss from pelvic fractures. Stabilization of fracture fragments prevents disruption of hematomas and possibly the dislodgement of hemostatic plugs.” [6].

During the 60ies to 80ies, some experts promoted pelvic ring stabilization as a treatment option in bleeding patients with pelvic fractures.

Evers et al. proposed first to perform a laparotomy for abdominal hemorrhage control (positive DPL) followed by mechanical stabilization of the pelvis (pneumatic antishock garment (PASG) or external fixation) in patients with observable pelvic hematoma, to control bleeding from bony fragments [21]. Angiography with selective arterial embolization was performed in patients with on-going hemodynamic instability or presence of an expanding retroperitoneal hematoma. In patients with only a positive count alone during diagnostic peritoneal lavage DPL, first pelvic mechanical fixation and angiography is recommended prior to laparotomy [21].

Flint et al. performed a selective approach to pelvic bleeding. Beside PASG (success rate 1/12), external fixation (success rate 3/4), selective angiography (success rate 0/1) and open reduction, internal fixation was performed in 37 patients. The overall mortality rate was 10% [25].

It was not until 1991, that Ben-Menachem et al. in their landmark paper on angio-embolization (AE), recommended additional immediate pelvic stabilization with an external fixator prior to AE [4].

In the late 80ies and early 90ies, mechanical stabilization of the disrupted pelvis became a standard in patients with pelvic fracture associated hemorrhage.

External fixation using a special clamp applied at the greater trochanter for unstable pelvic ring injuries was introduced in 1937 by Vorschütz (“Schraubzwinge”) [90]. Richter, in 1964, described a strap-type clamp to address posterior open book pelvic ring injuries [70]. In 1991, Reinhold Ganz introduced a rectangular clamp acting at the posterior pelvis for mechanical compression [29].

Application of the pelvic C-clamp offers a distinct biomechanical advantage over simple external fixation. Direct and improved stabilization of the posterior pelvic ring was achieved and provided the basis for effective pelvic tamponade [29, 65].

Since the early 1990s, the pelvic C-clamp came into routine use for emergency fixation of the pelvic ring (overview in: [28]), especially in European centers.

Introduction of the pelvic C-clamp in 1991 revolutionized the mechanical stabilization of the posterior pelvis

## Pelvic packing/tamponade

Spencer and Robinson controlled pelvic bleeding by dissecting the pubic symphysis in a patient with bilateral pubic rami fractures and thus approaching and treating retroperitoneal hemorrhage [77]. Probably they were the first to perform an extraperitoneal transsymphyseal approach to control retroperitoneal pelvic bleeding.

Lawson et al. performed an extraperitoneal approach to the true pelvis for retroperitoneal bleeding control. They reported on a case with on-going hemodynamic instability. The patient had bilateral pubic rami fractures and a right type B SI-joint injury [47].

A “long right paramedian incision.” was performed and “a large paravesical haematoma of about one and a half litres was evacuated, after which profuse arterial bleeding was seen to come from a vessel overlying the fracture of the right ilium. After further dissection the bleeding was seen to be due to an incomplete division of the right superior gluteal artery. The artery was divided, both ends were ligated and additional bleeding points in the fractured bone were occluded with wax.”.

Lawson et al. were the first to perform a longitudinal paramedian extraperitoneal approach to control retroperitoneal pelvic bleeding [47].

In the 60ies, some surgeons performed a direct extraperitoneal approach to retroperitoneal pelvic bleeding.

It was until 1979 that the first detailed report was published for extraperitoneal pelvic packing (“After incision, haematomata were evacuated and bleeding controlled by local tamponade.”) by Riska et al. from Helsinki, Finland [71].

They reported “on the operative management of 42 patients with comminuted pelvic fractures associated with massive haemorrhage”, treated between 1970 and 1974.

Several approaches were used to address the pelvic retroperitoneal region:

- mid-line incision.
- Pfannenstiel incision.
- anterior iliac crest-femoral incision.
- posterolateral incision.
- paramedian incision.

As an additional treatment modality, “In severe cases temporary compression of the abdominal aorta was necessary, and this procedure was best accomplished through a mid-line incision, which was extended proximally to allow retraction of the small intestine. Massive bleeding was found to be from arteries or the large veins. Bleeding from the exposed bone at fracture sites was insignificant.” [71]. Angiography was characterized disadvantageous as it was considered time consuming.

Riska et al. first reported in detail operative techniques to control pelvic bleeding within the pelvic region, but without additional stabilization of the pelvis [71].

It was the Hannover group that discussed the concept of pelvic packing in detail in the early 90ies. Bosch et al. proposed pelvic packing in diffuse bleeding from the dorsal venous plexus without describing the technique or a general treatment concept [5].

Tim Pohlemann et al. in 1993 first proposed the combined concept of mechanical posterior pelvic ring stabilization using the pelvic C-clamp, introduced by Reinhold Ganz in 1991 [29], followed by direct extraperitoneal packing of the presacral and paravesical plexus [87]. A detailed description of the technique of extraperitoneal pelvic packing was published in 1995 in English language [66].

The Hannover group (Pohlemann, Tscherne, Gänsslen) first described a concept of immediate mechanical stabilization of the posterior pelvic ring in completely unstable pelvic ring injuries (type C) followed by extraperitoneal pelvic packing in hemodynamically unstable patients or patients in extremis in the early and mid 90ies.

During the following years, this concept became standard in many European hospitals [19, 20, 60, 65].

In 1989, Otmar Trentz discussed several options to control pelvic bleeding [85]. It was stated that especially bleeding from venous plexus can sufficiently be managed by surgical tamponade. Even “open tamponade” during laparotomy can be performed with packing along the psoas splint and paracolic areas. Stabilization of the pelvic ring creates an abutment for the tamponade. Temporary external fixation and definitive internal fixation were suggested [85]. The Zürich group did not describe the sequence or type of stabilization.

In 2000, Harald Tscherne and his group reported on 15 patients in extremis treated according to the Hannover concept and described the technique again [88]. According to the TRISS method, a survival rate of 17.5% was expected, but the observed survival rate was doubled to 33.3%.

The combined approach to the exsanguinating pelvic injury with initial posterior mechanical stabilization using the pelvic C-clamp, followed by extraperitoneal pelvic packing became the standard of care for patients in extremis in the late 90ies.

In between, this concept was published in several leading trauma surgery textbooks. Currently, the concept of initial mechanical stabilization of the disrupted pelvis, followed by pelvic extraperitoneal packing is recommended in several clinical practice guidelines of many societies [7, 12, 53].

## The relevance of pelvic packing today–a literature review

Preperitoneal packing attempts to tamponade the venous sources of intrapelvic bleeding, and in case of tight packing, it may also play a role in arterial hemorrhage control [57].

A recent meta-analysis indicates that a treatment protocol including PP significantly reduces mortality and transfusion requirement before intervention in pelvic fracture patients with hemodynamic instability [49].

An electronic literature search was performed using Pubmed, the Cochrane library and Google Scholar. The timeline spanned from 2007 to 2023. A various combinations of the following keywords was used: “pelvic packing”, “pelvic tamponade”, “tamponade”, ”preperitoneal packing”.

In a scoping review, data on the identified literature was analyzed regarding demographic data, frequency, indications, data on hemodynamic instability, pelvic ring classification, transfusion requirements, time management, secondary procedures, hospital data, morbidity and mortality in patients with performed pelvic packing (PP).

### Demographic data

Data on 1001 patients with PP [8, 9, 11, 16, 26, 34, 36, 37, 39, 43, 44, 50, 51, 58, 61, 72, 74, 76, 80, 83, 91] are reported.

The male/female ratio was 2.15:1 [8, 9, 11, 16, 26, 34, 36, 39, 43, 44, 50, 51, 58, 72, 74, 76, 83, 91], the average age was 46.8years [8, 9, 11, 16, 26, 34, 36, 39, 43, 44, 50, 51, 58, 72, 74, 76, 83, 91], the mean Injury Severity Score (ISS) was 34.1 points [8, 9, 11, 16, 26, 34, 36, 37, 39, 43, 44, 50, 51, 58, 61, 72, 76, 80, 83, 91] and 84.3% had associated injuries PP [11, 16, 26, 34, 72, 76, 83].

### Frequency of pelvic packing

The mean frequency of PP in an overall group of 1% (864/85386 patients; range: 0.45–6.0%) [8, 9, 11, 14, 16, 26, 34, 37, 44, 50, 58, 61, 72, 76, 80, 83, 91]. In a nationwide analysis of 67,846 patients with blunt pelvic trauma, 307 patients (0.45%) had pelvic packing [58]. In reported hemodynamically unstable patients, PP is performed in average in 14.2% (202/1425 patients; range: 5.6–51.7%) [11, 15, 16, 36, 91].

Overall, pelvic packing is rarely necessary in the treatment of unstable pelvic ring fractures. Gaski et al. reported on 648 patients with Tile type B and C injuries of the pelvis, treated between 2002 and 2012 in Oslo, Norway [30]. Overall, in 4,17% PP was performed.

### Indications

Based on the literature, no clear concept regarding indication can be deduced. Several individual criteria were used to initiate PP. Hemodynamic and fracture-specific criteria can be distinguished.

- hemodynamic criteria: persistent systolic blood pressure (sBP) < 90 mmHg despite fluid and/or fluid resuscitation (2 L cristalloids, 2 PRBC) and/or BD < − 5mmol/l; ATLS class III to IV hemodynamic instability [8, 9, 11, 16, 26, 34, 36, 37, 39, 42, 50, 51, 61, 72, 76, 80, 83, 91].
- fracture-specific criteria: unstable pelvic ring injury (Tile type B or C) pelvic stabilization (anterior external fixator, pelvic C-clamp, pelvic binder) before [11, 16, 36, 91] or after PP [61].

Interestingly, only few reports favored the concept of mechanical stabilization first as a prerequisite for PP. The majority of reports only performed PP.

### Hemodynamic instability

Data on hemodynamic instability are available from 824 patients [8, 9, 11, 16, 26, 34, 37, 39, 43, 44, 50, 51, 61, 72, 74, 76, 80, 83, 91].

The average initial systolic blood pressure was 74.25 mmHg and the heart rate was 114.9/min [8, 9, 16, 34, 37, 39, 43, 51, 72, 80, 83, 91], representing a shock index of 1.55.

The first mean hemoglobin value after admission was 8.74 mg/dl [34, 39, 72, 76, 80, 83], the mean base deficit was − 10.34mmol/L [8, 9, 11, 16, 37, 43, 61, 74, 80, 83, 91] and the lactate value was 5.13mmol/L [43, 44, 51, 61, 76].

In six studies adequate data were available regarding numbers of PRBC from admission to PP [8, 50, 61, 80, 83, 91]. The average amount of PRBC was 5.1 (range: 2–11.8 PRBC until PP started).

The mean 24-hour transfusion requirement was 17.2 PRBC (range: 11–29 PRBC) [11, 16, 37, 39, 43, 50, 61, 80, 83, 91].

### Fracture classification

PP was performed in all fracture types. In six studies fractures were classified according to the AO/Tile classification [11, 26, 34, 50, 58, 83]: 17.2% type A injuries, 34.9% type B-injuries, 46.8% type C-injuries and 1.1% acetabular fractures.

In five studies, adequate data on the Young/Burgess classification were available [8, 39, 51, 61, 91]: 38% APC-injuries, 46% LC-injuries, 11.4% VS-type-injuries and 4.6% CM-fractures. Within the APC-group, there were 6% APC-1, 35.3% APC-2 and 58.7% APC-3 injuries. Within the LC-group, there were 24.8% LC-1, 41% LC-2 and 31.2% LC-3 injuries.

## Timing

The average time from admission until start of PP was 71.8 min (range: 36.25–96.8 min) [34, 37, 43, 50, 72, 80, 83]. The median time interval was 53 min (range: 35–77.5 min) [8, 39, 58, 61, 91]. The procedure time was stated in two studies: 23.3 min [43] and 60 min [50].

## Secondary procedures

The mean rate of secondary angio-embolization procedures was 36.4% [8, 9, 16, 34, 36, 37, 39, 50, 51, 58, 61, 74, 76, 83]. Of these, in 32.1% positive findings were reported [8, 34, 37, 51, 74, 83], which is an overall rate of positive findings in 11.7%.

The average time until embolization was 526.5 min (8 h:46 min; range: 165–636 min) [8, 9, 16, 37, 51, 61].

## Hospital data

Data for length of mechanical ventilation were available in five studies [8, 9, 16, 44, 91]. The average time of mechanical ventilation was 11 days (range: 6.5–14days). The average time on ICU was 13.7 days (range: 4.3–18 days) [8, 9, 16, 37, 44, 50, 51, 91] and the mean length of stay in the hospital was 25.3 days (range: 20.5–58 days) [8, 9, 16, 37, 51, 91].

## Complications-infections

Data on postoperative infection are reported by several aurthors. Papkostidis et al. performed a systematic review based on three studies. They reported a pooled estimation of infection of 35%. It must be considered, that 19.6% of these patients had open pelvic fractures [62].

Analysis of the literature from 2007 until 2023 reveals a much lower mean infection rate of 11.1% [8, 9, 16, 34, 37, 39, 44, 50, 58, 61, 76, 83, 91].

Burlew et al. observed 11.7% pelvic infections after PP. It has to be considered, that in 2/3 of these patients an open pelvic fracture or a severe perineal degloving injury was present [8]. Kim et al. reported on 34.1% pelvic infections. Risk factors were repacking and additional combined bladder-urethra injury [42]. Reitano et al. reported a rate of 8.3% [68].

## Complications-thromboembolism

The rate of thromboembolism after PP showed an average of 11.3% [34, 39, 44, 50, 61]. Heelan et al. analyzed 79 patients who had PP. 17 patients, who initally survived the first hours, had DVT, 6 had pulmonary embolism and 4 patients had both. All patients had external fixation or pelvic binder prior to PP but no data were available regarding definitive treatment nor time of diagnosis [36].

## Mortality

The overall mortality in the group of patients requiring PP was in average 27.6% (range: 7.1–50%) [8, 9, 11, 16, 26, 34, 36, 37, 39, 44, 50, 51, 58, 61, 72, 74, 76, 80, 83, 91].

## Treatment reality 2008–2023

The American Association for the Surgery of Trauma (AAST) analyzed 1339 patients with pelvic fractures from11 Level I trauma centers [15]. Of these, 178 patients (13.3%) were in shock/hemodynamically unstable.Emergency stabilization and bleeding treatment in all 1339 patients consisted of: 10,5% pelvic binder application, 9,6% angiography, 5,7% angioembolization, 2,6% EPP, 0,4% REBOA. In the hemodynamic unstable patient group, 18,5% had pelvic binder application, 24,7% had angiography and 16,9% had angioembolization.

In a multicenter study from New Zealand and Australia, 217 trauma patients from 11 major trauma centers with a major pelvic fracture (AIS pelvis ≥ 3) and hemodynamic instability were analyzed [89]. Unstable pelvic fractures were seen in 51.6% of patients. Overall, the rate of noninvasive pelvic stabilization in the ER were 28.6% (bed sheets 24.4%, pelvic binders in 3.3% and external fixators in 0.9%).

Hemorrhage Control was performed by 26.7% pelvic angiography, of these in 8.6% within 90 min. Emergency fixation techniques included external fixation (62/88, 70.5%), anterior plating (6/88, 6.8%), posterior screws (6/88, 6.8%), a combination of these methods (11/88, 12.5%), and pelvic C-clamp application (3/87, 3.4%).

In three regional Trauma Centers from Korea, 157 patients with haemorrhagic shock due to pelvic fracture (AIS ≥ 4, Tile type C) were analyzed retrospectively [41]. Pelvic packing was performed in 56,7% of patients. A pelvic external fixator was applied in only 12,7% and a pelvic binder in 54,8%. According to the fracture type, pelvic packing was performed in 62,5% after APC-injuries, in 46,5% after LC-injuries and in 73,3% after VS-injuries. Interestingly, the LC-group consisted of 16 LC-1 injuries which are normally not AIS grade 4 injuries.

Klingebiel et al. performed a questionnaire analysis about standard practice in the treatment of unstable pelvic ring injuries [45]. Emergency fixation using an external fixator was often used in 71.7%, while the pelvic C-clamp was rarely used (23.8%). For pelvic hemorrhage control, PP was often or always used in only 24.9%, angioembolization in 21.4% and REBOA in 7.6%.

Controversy exists over the optimal sequence of mechanical stabilization and hemorrhage control with PP. The main potential advantage with PP is earlier hemorrhage control, by overcoming the delay in accessing AE [37].

Pelvic compressive binding should be placed routinely in patients with suspected pelvic lesions before performing PPP [59]. An associated bone stabilization with a C-clamp or traditional external fixator can be performed to replace the pelvic binder [59]. In a network analysis, Tang et al. confirmed this concept of mechanical stabilization first before laparotomy or packing is considered. Angioembolization was indicated only complementary [81].

Monchal advised, that in patients with additional necessity of a laparotomy incision the incision for laparotomy and PP should be separated to „maintain the tamponade effect as long as possible“ [59].

### Studies comparing PP and AE or REBOA

Few studies compared PP and AE [37, 43, 50, 61, 80]. Overall, patients with PP were more severely injured (ISS), while hemodynamic parameters, e.g. base deficit (BD) and initial systloic blood pressure (sBP) were not different. Time until start of angioembolization was 1 h delayed compared to PP. Despite the lower ISS a higher mortality was observed after AE. Possibly, the delay in hemorrhage control may be responsible for this difference (average values; Table 1).

**Table 1:** Comparison of patients with PP and AE

Parameter	PP	AE
ISS	43.5 points	37.7 points
sBP	82.4mmHg	80.3mmHg
BD	− 10mmol/L	− 9.8mmol/L
Time to procedure	76.8 min	139.1 min
Mortality	25.2%	31.5%

In a systematic review and meta-analysis of 18 studies with a total of 579 patients, of which 402 were treated with PPP and 177 with angioembolization, it was reported that 27% of patients treated with PP did not achieve bleeding control and required subsequent angioembolization [57]. Patients with PP had a lower overall mortality rate (23% vs. 32%), a higher ISS (41 vs. 36 points) and a shorter time to intervention (60 min vs. 131 min).

Mikdad et al. compared 307 patients with primary PP with 113 patients who received REBOA [58]. A propensity score matching was performed resulting in 102 patients in each group. Both groups were comparable regarding age, gender, hemodynamic parameters, and ISS. The overall mortality rate was higher after REBOA (52% vs. 37.3%) and the 24-hour mortality rate was higher (32.4% vs. 17.7%).

Asmar et al. matched 52 with PP and 52 with REBOA [2]. 24-hour mortality was 25% vs. 14%), in-hospital mortality (44% vs. 29%) and 4-hour transfusion rate (15 vs. 10) were lower in the REBOA group. Additional external fixation was performed in 16% vs. 13%. In this study, REBOA was associated with better outcomes.

### Recommendations from clinical practice guidelines

At the First Italian Consensus Conference, a statement agreed that PP is effective and proposed an algorithm in which PP is performed prior to angiography [53].

World Society of Emergency Surgery (WSES) guidelines from 2017 recommend that PP should always be considered for patients with pelvic-fracture-related hemodynamic instability, and that maximum effectiveness can be achieved when it is combined with external fixation [12].

According to North American guidelines angiography remains the mainstay of therapy [7, 17, 82, 84]. However, surgeons in North America seem to be more in favor of angiography and embolization [7, 82].

## Conclusion

The indication for pelvic packing is still the hemodynamically unstable patient with an unstable pelvic ring injury. The initial treatment concept therefore is to mechanically stabilize the pelvis including the closed reduction of the pelvic ring followed by pelvic packing, as mechanical stability is the prerequisite for effective tamponade.

In the control of massive bleeding from internal iliac artery or its main branches, PP-procedure can facilitate early control of retroperitoneal bleeding and provide crucial time for more selective management of hemorrhage with AE. PP and AE play complementary roles in the control of rare exsanguinating bleeding from large pelvic arteries.

The potential risk of pelvic cavity infection seems to be predominantly associated with open fractures and fractures with a significant soft-tissue injury („complex pelvic trauma“) and in some cases with the PP-procedure.
